# Usefulness of Inflammation-Based Prognostic Scores in Patients with Surgically Treated Pancreatic Ductal Adenocarcinoma

**DOI:** 10.3390/jcm10245784

**Published:** 2021-12-10

**Authors:** Sarang Hong, Dae Wook Hwang, Jae Hoon Lee, Ki Byung Song, Woohyung Lee, Bong Jun Kwak, Yejong Park, Song-Cheol Kim

**Affiliations:** Division of Hepatobiliary and Pancreatic Surgery, Department of Surgery, Asan Medical Center, University of Ulsan College of Medicine, Seoul 05505, Korea; 8thofnovember@hanmail.net (S.H.); gooddr23@naver.com (J.H.L.); mtsong21c@naver.com (K.B.S.); ywhnet@gmail.com (W.L.); iio1000@nate.com (B.J.K.); blackpig856@gmail.com (Y.P.); drksc@amc.seoul.kr (S.-C.K.)

**Keywords:** pancreatic ductal adenocarcinoma, modified Glasgow Prognostic Score, neutrophil-lymphocyte ratio, platelet-lymphocyte ratio, prognosis

## Abstract

In this study, we evaluated the prognostic value of inflammation-based prognostic scores in patients undergoing curative surgery for pancreatic ductal adenocarcinoma (PDAC). A retrospective analysis was conducted for 914 patients undergoing curative surgical resection for PDAC between January 2011 and April 2016. Inflammation-based scores of modified Glasgow Prognostic Score (mGPS), neutrophil-lymphocyte ratio, and platelet-lymphocyte ratio were assessed. mGPS was classified as high (1 or 2) or low (0). Median age was 63 (range, 33–88) years; 538 patients (58.9%) were male. A high mGPS was independently associated with poor overall survival (OS) and disease-free survival (DFS) (median OS: 25.4 months vs. 20.4 months, *p* = 0.001; median DFS: 11.6 months vs. 9.3 months, *p* = 0.002), poor OS in patients with TNM stage I PDAC (44 months vs. 24.8 months, *p* = 0.001), and poor OS and DFS in patients with tumors located at the pancreatic head or uncinate process (OS: 25.4 months vs. 20.4 months; *p* = 0.007, DFS: 11.4 months vs. 8.87 months; *p* = 0.005). Preoperative mGPS was a significant prognostic factor for PDAC after curative resection; thus, mGPS can be a useful prognostic predictive factor in patients with TNM stage I PDAC, especially for tumors located at the head and uncinate.

## 1. Introduction

Pancreatic ductal adenocarcinoma (PDAC) is an aggressive disease with the worst prognosis of all gastrointestinal malignancies, even after curative resection [1,2,3,4,5]. Curative surgical resection is possible in only a minority of cases due to advanced disease status at the initial diagnosis. Furthermore, despite surgical resection, recurrence occurs in about 70% of patients [3,4,6,7]. Various factors have been advocated as prognostic indicators in patients with resected PDAC, such as tumor size, lymph node metastasis, resection margin status, perineural invasion, vascular invasion, and serum carbohydrate antigen 19-9 (CA 19-9) level [8,9,10,11].

Tumor progression and patient survival outcomes depend on both tumor biology and host-related factors. Various reports have described the systemic inflammatory markers affecting cancer survival since Virchow first proposed the link between inflammation and cancer in 1863 [12]. Several systemic inflammatory response markers, such as Glasgow Prognostic Score (GPS), modified GPS (mGPS), platelet-lymphocyte ratio (PLR), and neutrophil-lymphocyte ratio (NLR), have been evaluated and suggested to be predictive of various cancer survival rates. The mGPS was demonstrated as prognostic in lung, gastrointestinal, and renal cancers, while the NLR was demonstrated as associated with survival in lung, colorectal, and ovarian cancers [13,14,15,16,17].

Although many systemic inflammatory response markers have been evaluated for their prognostic value for PDAC, studies have shown different results [2,9,10,14,18,19,20,21]. Moreover, even if surgical resection provides the only chance of cure, given that the postoperative outcome remains poor and pancreatic surgery usually carries morbidity and mortality, preoperative markers that could serve a role in high-risk patients avoiding surgery are worth evaluation [22].

This study aimed to evaluate the value of inflammation-based prognostic scores, including mGPS, NLR, and PLR, in patients undergoing curative surgery for PDAC and suggest it as a parameter predictive of postoperative survival.

## 2. Materials and Methods

### 2.1. Patients

The 1172 patients who underwent surgical resection for pancreatic tumors at Asan Medical Center between January 2011 and April 2016 were identified, and their medical records were retrospectively reviewed, after receiving institutional review board approval (No. 2021-1176). Among them, 258 patients who underwent palliative surgery, had tumors other than PDAC, or for whom data related to inflammation-based scores were unavailable were excluded; thus, a total of 914 patients were enrolled. The patients’ demographic information, including age and sex, were retrieved. Disease status and preoperative laboratory profiles, especially serum C-reactive protein (CRP) level, serum albumin level, CA 19-9 level, neutrophil count, lymphocyte count, and platelet count, were also retrieved. Cancer pathological stage was classified according to the Tumor Node Metastasis (TNM) Classification of Malignant Tumors, 8th edition, of the American Joint Committee on Cancer (AJCC).

Pancreaticoduodenectomy (pylorus-preserving (PPPD) or pylorus-resecting (PrPD)) was performed for tumors located in the pancreas head or uncinate. Distal pancreatectomy (DP) was the standard surgical procedure for cancer in the pancreatic body or tail. Neoadjuvant chemotherapy was introduced in 2007 in our center, and adjuvant chemotherapy was administered based on patients’ general condition [1,23,24]. All specimens were reviewed by pathologists. Pathologic characteristics included tumor size, resection margin status, lymph node metastasis, and differentiation.

### 2.2. Inflammation-Based Prognostic Scores

Preoperative blood samples were collected at the time of admission, prior to the endoscopic or biliary drainage procedure. Inflammation-based prognostics scores of mGPS, NLR, and PLR were assessed using lab profiles. The mGPS was calculated for every patient, and they were classified into three groups accordingly. Patients with an elevated CRP level (>10 mg/L) and hypoalbuminemia (<35 g/L) were classified as mGPS 2, those with only an elevated CRP level were classified as mGPS 1, and those with neither of these factors were classified as mGPS 0. We divided patients into two groups based on mGPS score: low (mGPS = 0) and high (mGPS = 1 or 2) [25,26]. NLR was calculated by dividing the neutrophil count by the lymphocyte count, while PLR was calculated by dividing the platelet count by the lymphocyte count. Cut-off values for the NLR and PLR were determined based on previous studies [2,21,27,28,29].

### 2.3. Statistical Analysis

Overall survival (OS) was defined as the time interval between the surgery and the date of death of any cause or the last follow-up visit. Disease-free survival (DFS) was defined as the time from the surgery to disease recurrence or death, whichever occurred first. The cut-off follow-up date was 17 July 2018. Survival was checked using hospital records and the national health insurance registry review. OS and DFS were estimated using the Kaplan-Meier method and compared using the log-rank test. We evaluated the correlation between clinicopathological values and OS or DFS using univariate and multivariate analyses. Univariate and multivariate analyses were performed using the Cox proportional hazard regression model and are presented as hazard ratios (HRs) with 95% confidence intervals (CIs). Categorical variables are presented as numbers and percentages. *p* values < 0.05 were considered statistically significant. All statistical analyses were performed using SPSS Statistics for Windows version 21.0 (IBM Corp. Armonk, NY, USA).

## 3. Results

### 3.1. Patient Characteristics

**Figure 1:**
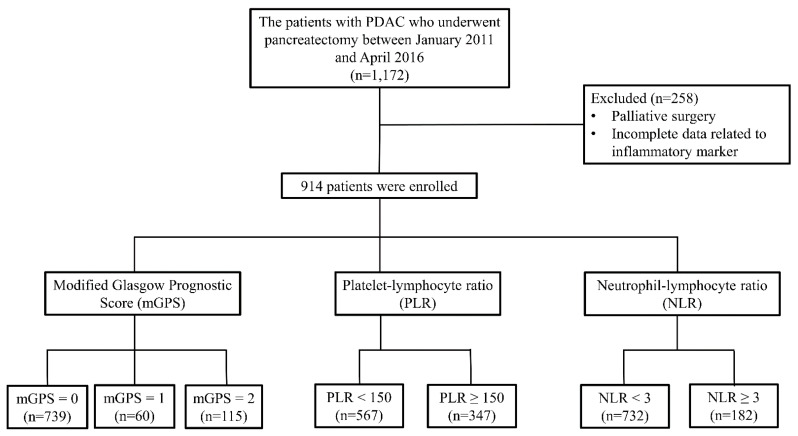
Flowchart of patient selection process. PDAC, pancreatic ductal adenocarcinoma.

Of the 1172 patients, 914 who underwent curative pancreatic surgery with complete preoperative laboratory profiles related to inflammation-based prognostic scores were included in this study; their demographics are summarized in Table 1. The median patient age was 63 (range, 33–88) years; there were 538 men (58.9%) and 376 women (41.1%). A total of 606 patients (66.3%) underwent the Whipple operation, PPPD, or PrPD; 257 (28.1%) underwent DP; and 51 (5.6%) underwent laparoscopic or open total pancreatectomy, whichever was more suitable. A total of 608 patients (66.5%) had an elevated preoperative serum CA 19-9 level (>37 U/mL). Tumors were staged according to the AJCC 8th edition of the TNM staging system: 297 (32.5%) as stage I, 439 (48%) as stage II, and 178 (19.5%) as stage III. A total of 739 patients (80.9%) were scored as mGPS 0, 60 (6.6%) as mGPS 1, and 115 (12.6%) as mGPS 2. A total of 732 patients (80.1%) were rated as having a low NLR (<3), while 182 (19.9%) had a high NLR (≥3). A total of 567 patients (62%) were rated as having a low PLR (<150), while 347 (38%) had a high PLR (≥150). 61 patients (6.7%) had neoadjuvant treatment, and 603 patients (66%) had adjuvant treatment, either chemotherapy alone or concurrent chemo-radiotherapy. A flowchart of the patient selection process is shown in Figure 1.

### 3.2. OS and DFS Based on Inflammation-Based Prognostic Scores

**Figure 2:**
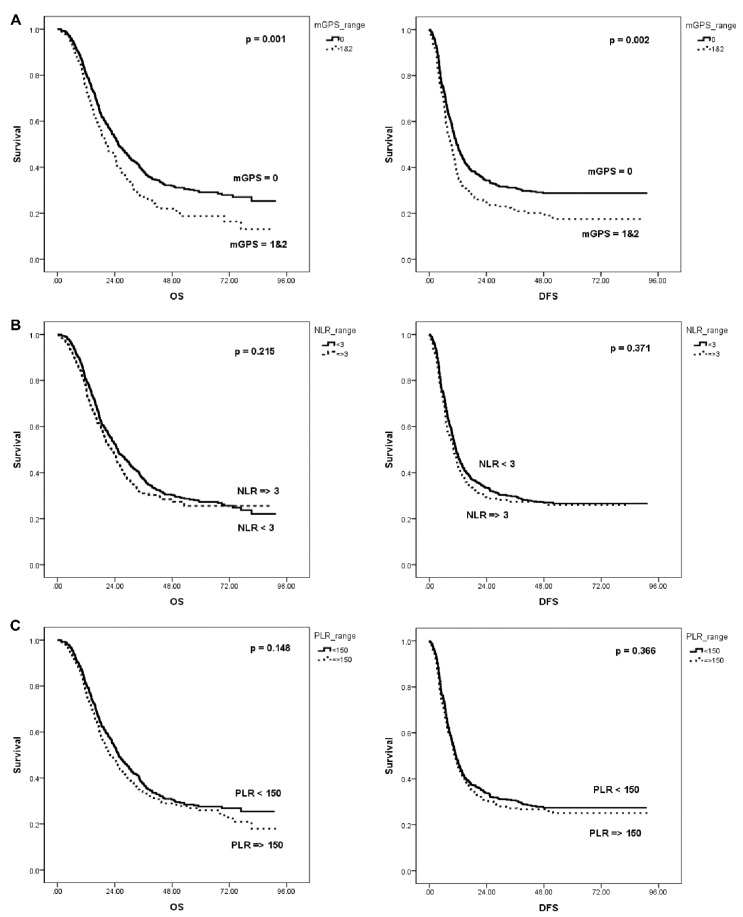
OS and DFS based on each inflammation-based prognostic score. (**A**) mGPS, (**B**) NLR, (**C**) PLR. OS, overall survival; DFS, disease-free survival; mGPS, modified Glasgow Prognostic Score; NLR, neutrophil-lymphocyte ratio; PLR, platelet-lymphocyte ratio.

Patients were divided into two groups according to the cut-off level of 3 and 150 for NLR and PLR, respectively. They were also grouped into two low (mGPS = 0) or high (mGPS = 1 or 2) groups. When evaluating the relationship between inflammatory prognostic scores and OS and DFS, mGPS was significantly associated with both OS and DFS. Patients with a low mGPS demonstrated significantly better OS than those with a high mGPS (median OS, 25.4 months vs. 20.4 months, *p* = 0.001). As with OS, patients with a low mGPS showed a significantly better DFS than those with a high mGPS (median DFS, 11.6 months vs. 9.3 months, *p* = 0.002) (Figure 2). However, there was no significant intergroup difference in OS and DFS by NLR (OS: high vs. low, 22.5 months vs. 25.2 months; *p* = 0.215, DFS: high vs. low, 10.2 months vs. 11.2 months; *p* = 0.371) and PLR (OS: high vs. low, 22.1 months vs. 25.6 months; *p* = 0.148, DFS: high vs. low, 10.8 months vs. 11.2 months; *p* = 0.366).

**Figure 3:**
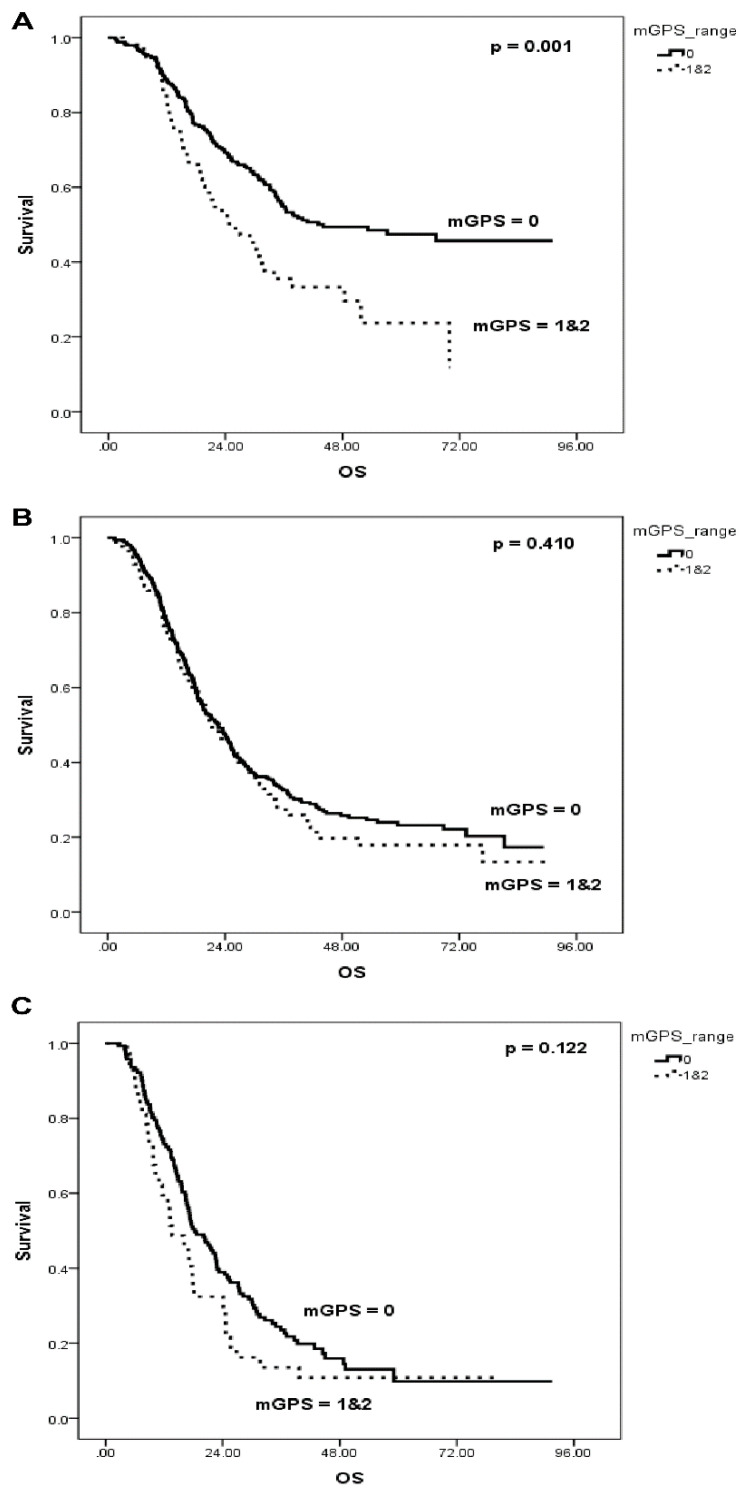
OS according to mGPS by TNM stage. (**A**) Stage I, (**B**) Stage II, (**C**) Stage III. OS, overall survival; DFS, disease-free survival; mGPS, modified Glasgow Prognostic Score; TNM, Tumor Node Metastasis.

OS curves were constructed for each TNM stage based on each inflammation-based prognostic score. For all TNM stages, OS did not differ between the low and high NLR groups or between the low and high PLR groups. However, a low mGPS was significantly associated with better OS than a high mGPS for stage I (44 months vs. 24.8 months, *p* = 0.001) but not for II/III (Figure 3).

The survival outcome based on each inflammatory marker was conducted by surgery type. For those undergoing the Whipple operation, OS and DFS were significantly different between the low and high mGPS groups (OS: 25.4 months vs. 20.4 months, *p* = 0.007; DFS: 11.4 months vs. 8.87 months, *p* = 0.005). However, for patients undergoing DP, OS was not significantly different between the low and high mGPS groups (27.2 months vs. 24.4 months, *p* = 0.340).

### 3.3. Univariate and Multivariate Analyses of OS

Table 2 shows the potential prognostic factors associated with OS. The factors demonstrating a potential association (*p* < 0.05) with OS in the univariate analysis were included in the multivariate analysis. Age older than 65 years (HR, 1.245; *p* = 0.007), large tumor size (HR, 1.989; *p* < 0.001), a positive resection margin (HR, 1.352; *p* = 0.001), lymph node metastasis (HR, 1.667; *p* < 0.001), poor differentiation (HR, 3.098; *p* < 0.001), and a high mGPS (HR, 1.268; *p* = 0.015) were significant prognostic factors for OS in the multivariate analysis. CA 19-9 level was a significant factor on the univariate analysis but not on the multivariate analysis (*p* = 0.287).

## 4. Discussion

In the present study based on a large cohort, patients with an mGPS rated as either 1 or 2 showed significantly poorer OS and DFS (*p* = 0.001 and *p* = 0.002) than those with an mGPS rated 0. A high mGPS was also significantly associated with a poor OS in patients with early pancreatic cancer, i.e., TNM stage I (*p* = 0.001). Previously, mGPS was demonstrated to affect survival, independent of stage, and showed significantly different survival rates between the stage I/IIa and stage IIb/III groups [19]. NLR and PLR, however, were not significant prognostic factors for OS or DFS at any stage. Similar to previous studies [5,10,11,30,31], tumor size, resection margin status, lymph node status, and histological differentiation were also significant risk factors associated with OS in the present study.

As is widely known, host-related factors such as inflammatory markers are reportedly prognostic factors in many solid cancers, such as esophageal, gastric, colorectal, and gallbladder cancers [20,25,32,33,34,35,36]. Among the several inflammation-based prognostic scores, mGPS, NLR, and PLR are commonly evaluated for prognostic prediction. However, due to uncertainty and diversity among studies [11,14,21,37], we evaluated their prognostic value in a large cohort of patients.

We analyzed survival outcome regarding surgery type, and OS and DFS were significantly different between the low and high mGPS groups (OS: *p* = 0.007; DFS: *p* = 0.005) among patients undergoing the Whipple operation but not distal pancreatectomy. The number of patients undergoing total pancreatectomy was too small to compare. Tumors located at the head or uncinate process often invade the bile duct or duodenum, resulting in biliary obstruction and cholangitis, which can elevate serum CRP levels. Patients enrolled in the present study underwent blood sampling before the biliary drainage, and we can infer that the elevated serum CRP level might have resulted from cholangitis. Since a high mGPS was demonstrated as a significant prognostic factor, managing systemic inflammation by preoperative biliary drainage and biliary infection control could improve the survival benefit [2], while preoperative biliary drainage might be a management modality.

Among several inflammatory prognostic markers, CRP is the most commonly studied, while mGPS was revealed to have prognostic value in cancer, independent of tumor site [13,38]. In cancer patients, nutritional and functional decline are associated with a poorer outcome, while a chronic inflammatory state is known to be linked with anorexia–cachexia syndrome. An elevated mGPS is associated with increased weight loss, poor performance status, increased comorbidities, and increased pro-inflammatory and angiogenic cytokines, all of which can lead to a poor outcome. Furthermore, the immune response facilitates tumor progression, and tumor cells can produce inflammatory chemical mediators, assisting tumor growth [12,39]. The present study’s findings emphasize the relationship between systemic inflammation and survival in patients with pancreatic cancer as well as the value of mGPS as a significant prognostic factor.

This study has some limitations. This was a retrospective and single-center study; thus, selection bias was unavoidable. However, this study included a large patient population of more than 900 patients. Second, as the optimal cut-off values for each inflammation-based prognostic score vary among studies, the values that we used for NLR and PLR might have affected the results. Since no single value has been reported as a definite cut-off value, further studies of optimal cut-off values in large cohorts are necessary.

In conclusion, as preoperative mGPS was a significant prognostic factor for PDAC after curative resection, it can be useful in patients with TNM stage I PDAC, especially for tumors located at the head and uncinate, to predict postoperative outcomes.

## Figures and Tables

**Table 1:** Patient demographics.

Characteristics	Patients, *n* = 914 (%)
Age, median	63 (33–88)
Sex	
Male	538 (58.9%)
Female	376 (41.1%)
Operation Whipple/PPPD/PrPD Distal pancreatectomy Total pancreatectomy	606 (66.3%) 257 (28.1%) 51 (5.6%)
CA 19-9 Normal Elevated (>37 U/mL)	306 (33.5%) 608 (66.5%)
T stage T1 and T2 T3 and T4	735 (80.4%) 179 (19.6%)
LN metastasis Absent Present	351 (38.4%) 563 (61.6%)
TNM stage (AJCC 8th) I II III	297 (32.5%) 439 (48%) 178 (19.5%)
Resection margin status Negative Positive	656 (71.8%) 258 (28.2%)
mGPS 0 1 2	739 (80.9%) 60 (6.6%) 115 (12.6%)
NLR <3 ≥3	732 (80.1%) 182 (19.9%)
PLR	
<150	567 (62%)
≥150	347 (38%)
Neoadjuvant treatment	
No	853 (93.3%)
Yes	61 (6.7%)
Ajuvant treatment	
No Yes	311 (34.0%) 603 (66.0%)

AJCC, American Joint Commission on Cancer; CA, carbohydrate antigen; mGPS, modified Glasgow Prognostic Score; LN, lymph node; NLR, neutrophil-lymphocyte ratio; PLR, platelet-lymphocyte ratio; PPPD, pylorus preserving pancreaticoduodenectomy; PrPD, pylorus resecting pancreaticoduodenectomy; TNM, Tumor Node Metastasis.

**Table 2:** Univariate and multivariate analyses of factors associated with OS.

Factors	No. of Patients	Univariate (P)	Hazard Ratio (95% CI)	Multivariate (P)
Age (years) <65 ≥65	515 399	0.027	1.245 (1.061–1.462)	**0.007**
Gender		0.120		
Male	538			
Female	376			
Tumor size (cm)		0.000		**0.000**
≤2	140			
>2 and ≤4	603		0.410 (1.089–1.828)	
>4	171		1.989 (1.482–2.668)	
RM		0.000		**0.001**
Negative	656			
Positive	258		1.352 (1.138–1.606)	
Lymph node metastasis		0.000		**0.000**
Absent	351			
Present	563		1.667 (1.399–1.987)	
Differentiation		0.000		**0.000**
Well D	104			
Moderate D	675		1.901 (1.414–2.556)	
Poor D	107		3.098 (2.175–4.413)	
CA 19-9 Normal Elevated	306 608	0.002		0.287
mGPS 0 1 and 2	739 175	0.001	1.268 (1.047–1.537)	**0.015**
NLR <3 ≥3	732 182	0.215		
PLR		0.149		
<150	567			
≥150	347			
Neoadjuvant treatment No Yes	853 61	0.814		
Adjuvant treatment No Yes	311 603	0.655		

CA, carbohydrate antigen; CI, confidence interval; mGPS, modified Glasgow Prognostic Score; NLR, neutrophil-lymphocyte ratio; OS, overall survival; PLR, platelet-lymphocyte ratio.

## Data Availability

Not applicable.
